# Adenosine A2A Receptors Mediate Anti-Inflammatory Effects of Electroacupuncture on Synovitis in Mice with Collagen-Induced Arthritis

**DOI:** 10.1155/2015/809560

**Published:** 2015-02-16

**Authors:** Qi-hui Li, Wen-xia Xie, Xiao-pei Li, Ka-te Huang, Zhong-heng Du, Wen-jie Cong, Long-hua Zhou, Tian-shen Ye, Jiang-Fan Chen

**Affiliations:** ^1^Department of Acupuncture, The First Affiliated Hospital of WenZhou Medical University, Wenzhou, Zhejiang 325000, China; ^2^Department of Pathology, The First Affiliated Hospital of WenZhou Medical University, Wenzhou, Zhejiang 325000, China; ^3^Department of Neurology, Boston University School of Medicine, Boston, MA 02118, USA

## Abstract

To study the role of adenosine A2A receptor (A_2A_R) in mediating the anti-inflammatory effect of electroacupuncture (EA) on synovitis in collagen-induced arthritis (CIA), C57BL/6 mice were divided into five treatment groups: *Sham-control, CIA-control, CIA-EA, CIA-SCH58261* (A_2A_R antagonist), and *CIA-EA-SCH58261*. All mice except those in the *Sham-control* group were immunized with collagen II for arthritis induction. EA treatment was administered using the stomach 36 and spleen 6 points, and stimulated with a continuous rectangular wave for 30 min daily. EA treatment and SCH58261 were administered daily from days 35 to 49 (*n* = 10). After treatment, X-ray radiography of joint bone morphology was established at day 60 and mouse blood was collected for ELISA determination of tumor necrosis factor alpha (TNF-*α*) levels. Mice were sacrificed and processed for histological examination of pathological changes of joint tissue, including hematoxylin-eosin staining and immunohistochemistry of A_2A_R expression. EA treatment resulted in significantly reduced pathological scores, TNF-*α* concentrations, and bone damage X-ray scores. Importantly, the anti-inflammatory and tissue-protective effect of EA treatment was reversed by coadministration of SCH58261. Thus, EA treatment exerts an anti-inflammatory effect resulting in significant protection of cartilage by activation of A_2A_R in the synovial tissue of CIA.

## 1. Introduction

Rheumatoid arthritis (RA) is a chronic, progressive, and disabling autoimmune disease characterized by synovial hyperplasia and progressive joint destruction. Its inflammatory pathological process is associated with synovial proliferation and secretion of high levels of proinflammatory mediators such as tumor necrosis factor-*α* (TNF-*α*) and interleukin-1 (IL-1). These inflammatory mediators activate several cell types including lymphocytes, neutrophils, macrophages, synovial fibroblasts (RASF), and chondrocytes [1]. Among these cells RASF are considered key cells [2] in driving the pathological processes. Furthermore, as a dominant cell type in the hyperplastic rheumatoid synovium, RASF play a key role in inflammatory cascade amplification, bridging innate and adaptive immunity [3]. Proinflammatory cytokines, such as TNF-*α* and IL-1, play an important role both in the continuous stimulation of RASF and in the crosstalk between RASF and other cell types in the synovium [4]. TNF-*α* is an instrumental cytokine that triggers inflammation and joint destruction. Overexpression of TNF-*α*, as achieved in the TNF transgenic mouse model, appears to be sufficient to initiate chronic synovitis, cartilage destruction, and bone erosion [5].

Traditional drug therapy for RA includes nonsteroidal anti-inflammatory drugs and disease-modifying antirheumatic drugs. All these drugs, however, tend to have significant side-effects (such as organ toxicity) and contraindications and result in drug dependence and tolerance. This means the treating physician must consider complementary therapies, hoping that such treatment will be safe and ameliorate the symptoms of RA. Therefore, exploration of new antirheumatic treatments with high efficacy and less toxicity are urgently needed. Acupuncture is one of the most commonly used complementary therapies for the treatment of RA in China, and some reports confirm that electroacupuncture (EA) showed favorable efficacy in treating RA [6–8]. Despite its long history and worldwide practice, the mechanism of acupuncture mediated effects on chronic inflammatory pain has not yet been fully elucidated. Recently, Goldman et al. advanced the research of acupuncture in mice and found a clear biological mechanism of acupuncture [9]. These investigators found that adenosine levels increased at the acupuncture site while pain was simultaneously ameliorated. However, mice lacking a key cellular receptor for adenosine did not show this response. Moreover, Takano and colleagues, using the same method in human subjects [10], reported that the interstitial adenosine concentration increased significantly during acupuncture and remained elevated for 30 minutes after the treatment.

Adenosine, a well known purine nucleoside, acts as a potent endogenous inhibitor of inflammatory processes in some tissues. Adenosine interacts with four G-protein-coupled adenosine receptors termed A1R, A_2A_R, A_2B_R, and A3R. Recent studies have pointed out the important role of adenosine in the process of inflammation in RA [11]. Several authors have reported that activation of A_2A_R inhibits TNF-*α* production in human peripheral blood mononuclear cells and suppresses the elevated levels of TNF-*α* and IL-1*β* in RA [12, 13]. Mazzon and colleagues also reported that intraperitoneal injection of the adenosine A_2A_R agonist, CGS21680, resulted in relief of arthritis symptoms in CIA mice and a decrease in release of TNF-*α* and IL-1*β*, pointing to a role of A_2A_R in CIA [14]. In addition, Mediero et al. reported that A_2A_R activation inhibits osteoclast formation* in vitro* and* in vivo* [15].

Liu et al. [16] found that the nonselective adenosine receptor antagonist, caffeine, inhibited the anti-inflammatory effect of electroacupuncture in CIA rats. In addition, Varani et al. [17] reported that low frequency, low energy pulsed electromagnetic fields upregulated the expression of A_2A_R in bovine chondrocytes and synovial fibroblasts. These results suggested that adenosine may represent a potential mediator of the therapeutic effect of EA. In order to further explore the potential role of adenosine in the mechanism of EA treatment of arthritis, we chose to investigate the role of A_2A_R in EA treatment of CIA.

## 2. Materials and Methods

### 2.1. Animals

Seventy male C57BL/B6 mice (4-5 weeks of age) were used for these studies. Animals were purchased from the Laboratory Animal Center of Silaike in Shanghai, China, housed in a controlled environment, and provided with standard rodent chow and water. Animal care was in compliance with regulations on protection of animals used for experimental and other scientific purposes. Ten mice were randomized into a* Sham-control* group (see below); all other mice were subjected to induction of CIA.

### 2.2. Induction of CIA

Chicken type II collagen (CII) was dissolved in 0.01 M acetic acid at a concentration of 2 mg/mL by stirring overnight at 4°C. Dissolved CII was frozen at −70°C until use. Complete Freund's adjuvant (CFA) was prepared by addition of* Mycobacterium tuberculosis* H37Ra (Chondrex, Redmond, WA, USA) at a concentration of 2 mg/mL. Before injection, CII was emulsified with an equal volume of CFA. CIA was induced as described [18]. Since the CFA was mixed with an equal volume of CII, the final concentration of* M. tuberculosis* present in the emulsion was 2.5 mg/mL (250 *μ*g per mouse as 100 *μ*L was injected). The final concentration of CII was 1 mg/mL (100 *μ*g per mouse as 100 *μ*L was injected). On day 1, sixty mice were injected intradermally at the base of the tail with 100 *μ*L of the emulsion. At day 21 after the first injection, mice received a second injection at the base of the tail close to the previous injection site.

### 2.3. Clinical Assessment of CIA

Development of arthritis in mice was evaluated every other day starting from day 18 after the first intradermal injection. Animals were scored for clinical signs of arthritis as follows: 0 = normal; 1 = swelling of at least one toe joint; 2 = swelling of paws; 3 = all foot paws with swelling but not involving ankle; 4 = all foot paws with swelling and involving ankle. The arthritis index for each mouse was calculated by adding up the 4 scores of individual paws, allowing a maximum score of 16 per mouse. CIA was regarded as induced when the score was higher than 1 in more than two joints or higher than 2 in more than one joint. Only mice with a score of ≥4 were included in any of the experimental groups.

### 2.4. Experimental Groups

Mice were divided into the following 5 groups.

*Sham-Control*. Mice were subjected to an intradermal injection at the base of the tail with 100 *μ*L of 0.01 M acetic acid instead of the emulsion containing 100 *μ*g of CII in CFA. These mice were only subjected to fixation using the bag fixation method but no other interventions were used during the testing process. 

*CIA-Control*. Mice were subjected to development of CIA and administered 200 *μ*L of 10% DMSO solution intraperitoneally (vehicle for SCH58261) every 24 h, starting from day 35 to day 49 (*n* = 10). 

**Figure 1:**
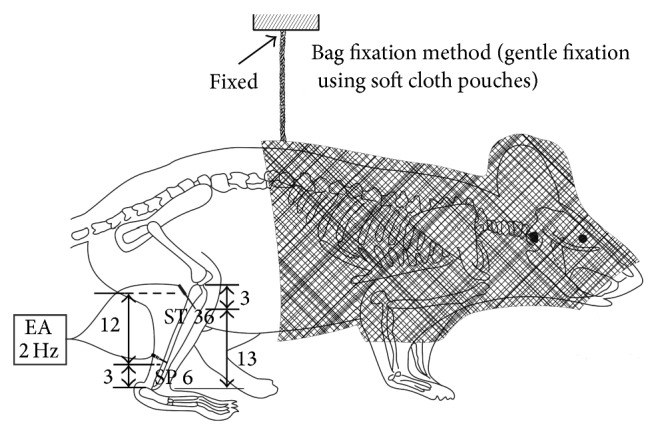
EA treatment of mice. Schematic drawing of bag fixation and electroacupuncture using the ST 36 and SP 6 points. After mice were immobilized in a soft cloth pouch, the mice in the* CIA-EA* group were electrically stimulated with a 2 Hz current in a rectangular wave form for 30 min and were treated with EA for 14 days starting on the 14th day after the primary immunization. Mice in the other groups were maintained in the same bags for 30 min without acupuncture.

*CIA-EA*. Mice were subjected to development of CIA and treated with EA. The mouse stomach point 36 (ST 36) and the spleen point 6 (SP 6) were determined according to Chinese Acupuncture and Moxibustion [19, 20]. An intradermal acupuncture needle (0.22 mm × 5.0 mm, a special type of acupuncture needle, Hua tuo Acupuncture, China) was inserted into ST 36 that is located longitudinally three body inches below the knee joint, transversely in the middle of the tibialis anterior muscle. Another needle was inserted into SP 6 that is located three body inches above the apex of the medial malleolus, behind the tibia. Both needles were inserted to a depth of 2 mm. An electrical stimulator was connected to the inserted acupuncture needles, and a continuous rectangular wave current (2 Hz, 0.07 mA, 0.3 ms) was applied to the needle for 30 min every 24 h starting from day 35 to day 49 (*n* = 10). EA treatment was administered to the left and right hind legs simultaneously (Figure 1).

*CIA-EA-SCH58261*. Mice were subjected to CIA, treated with EA, and were administered SCH58261 (5 mg/kg intraperitoneally) before EA as described above, every 24 h starting from day 35 to day 49 (*n* = 10). 

*CIA-SCH58261*. Mice were subjected to CIA but not treated with EA and were administered SCH58261 (5 mg/kg intraperitoneally) as described above, every 24 h starting from day 35 to day 49 (*n* = 10).

### 2.5. Bag Fixation Method

We immobilized the mice during electroacupuncture treatment. This method of gentle fixation used soft cloth pouches to cover and restrain approximately the anterior half of the animals including the face. Compared with common rigid restrainers, these pouches allowed mice more freedom to move body and limbs and created an environment which was dark and warm, minimizing the impact of stress during the experimental treatment. All groups were restrained for 30 minutes a day during the period of treatment (Figure 1).

### 2.6. Radiography

At day 60 after the first injection, all mice were anesthetized with 4% chloral hydrate (0.1 mL/10 g, intraperitoneally). Mice were placed on a radiographic box 100 cm from the X-ray source (dental X-ray machine). Radiographic analysis of normal and arthritic rat hind paws was performed (Philips X12, Germany) with an exposure of 40 kW for 0.01 s. An investigator blinded to the treatment regimen performed the radiograph scoring. The following radiographic criteria were considered: score 0, no bone damage; score 1, tissue swelling and edema; score 2, joint erosion; score 3, bone erosion and osteophyte formation. The radiograph index was calculated by adding up the scores of the two hind paws of each mouse. The results were expressed as the mean value for each mouse.

### 2.7. Measurement of TNF-*α*

TNF-*α* levels were determined in plasma. Plasma was collected from the blood in the orbit of the mice on day 61. Briefly, TNF-*α* was measured using a commercial ELISA according to the manufacturer's instructions (R&D Systems, Minneapolis, MN). The values were normalized for total protein concentration. The sensitivity of these assays was 1 pg/mg of total protein.

### 2.8. Histological Examination

All mice were sacrificed under anesthesia (4% chloral hydrate 0.1 mL/10 g, intraperitoneally); and ankles and knees were removed and fixed in 4% paraformaldehyde solution for 24 hours. Knee joints were decalcified in 10% EDTA (pH 8.0, Sigma, USA) for 2-3 weeks and embedded in paraffin. Paraffin-embedded sections of joints were sectioned and stained with hematoxylin and eosin for histologic assessment. Arthritis severity in histologic samples was determined by cumulative assessment of synovial inflammation. Arthritis damage (histological damage score) was evaluated and scored by an investigator blinded to the treatment regimen. The following morphological criteria were considered: score 0, no damage; score 1, edema; score 2, presence of inflammatory cells; score 3, bone resorption.

### 2.9. Immunohistochemical Localization of A_2A_R

Ankle joints were trimmed and placed in decalcifying solution for 24 h and 8 *μ*m sections were prepared from paraffin-embedded tissues. After deparaffinization, endogenous peroxidase was quenched with 0.3% H_2_O_2_ in 60% methanol for 30 min. The sections were permeabilized with 0.1% Triton X-100 in phosphate buffered saline (PBS) for 20 min. Nonspecific adsorption was minimized by incubating the section in 2% normal goat serum in PBS for 20 min. Endogenous biotin or avidin binding sites were blocked by sequential incubation for 15 min with avidin and biotin (sensitizing agent, PK-4002, Vector laboratories, USA). Sections were incubated overnight with an anti-mouse polyclonal antibody directed at A_2A_R (1 : 200 in PBS, vol/vol). Controls included buffer alone and nonspecific purified mouse IgG. Specific labeling was detected with biotinylated pan-specific antibody (goat anti-mouse IgG; DBA). Immunocytochemistry photographs (*N* = 5) were assessed by densitometry using an imaging densitometer with computer software. Each section was examined at high magnification (400x) and was tested only once. Results are expressed as means ± SEM for ten mice in each group.

### 2.10. Statistical Analysis

All data are expressed as the mean ± standard error (SEM). Data were analyzed by two-way analysis of variance followed by post hoc evaluation. If the variance was not neat, we used Tamhane's T2 method instead. All statistical analyses were performed with the SPSS software, version 16.0. In all cases, *P* values of less than 0.05 were considered significant.

## 3. Results

### 3.1. Induction of Arthritis in C57BL/B6 Mice

**Figure 2:**
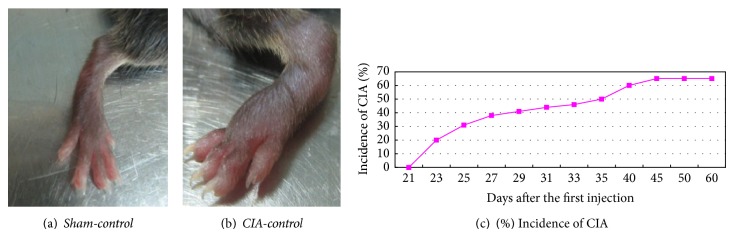
The CIA model, clinical symptoms and time course. (a) No clinical signs of CIA were observed in* Sham-control* mice. (b) CIA developed rapidly in mice immunized with CII, with clinical signs such as periarticular edema and erythema.

The C57BL/B6 mice developed clinical signs of arthritis with an incidence of 65% by day 45 after primary immunization. C57BL/B6 mice exhibited clinical signs of arthritis equivalent to scores covering the full range from 1 to 16 with some limbs showing severe swelling of the footpad, ankle/wrist joint, and digits. The clinical appearance of the swollen joints, the range of severity, and the progression to severe swelling and ankylosis were similar to those observed in other strains of mice (Figure 2).

### 3.2. Results of Radiographic Examination

**Figure 3:**
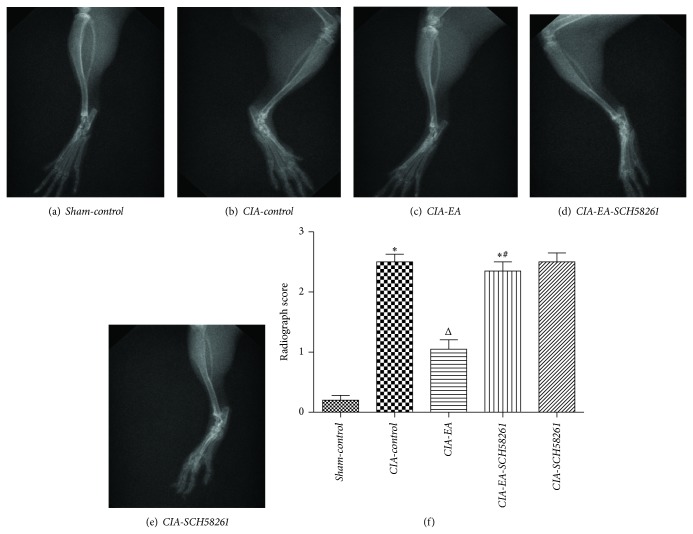
Radiography of hind paws with CIA. Radiographic assessment of bone damage in the tibiotarsal joints of mice with CIA. There is no evidence of bone resorption in joints of mice in the* Sham-control* group (a, f). Hind paws from* CIA-control* mice showed bone resorption (b, f).* CIA-EA* mice showed less bone resorption than* CIA-control* mice (b, c). There was no significant decrease of the radiographic score of* CIA-EA-SCH58261* mice (d, f) and* CIA-SCH58261* mice (e, f) compared to* CIA-control* mice. Values are means ± SEM of 10 animals for each group. ^*^ *P* < 0.01 versus* Sham-control*. ^Δ^ *P* < 0.01 versus* CIA-control*. ^#^ *P* < 0.01 versus* CIA-EA*.

Radiographic examination of knee and ankle joints and digits from* CIA-control* mice at 60 days after CII immunization revealed bone erosion. Significantly less bone erosion was observed in* CIA-EA* mice. There was no significant difference between the radiographic score of either* CIA-EA-SCH58261* mice or* CIA-SCH58261* mice and* CIA-control* mice (Figure 3, Table 1).

### 3.3. Histological Evaluation

**Figure 4:**
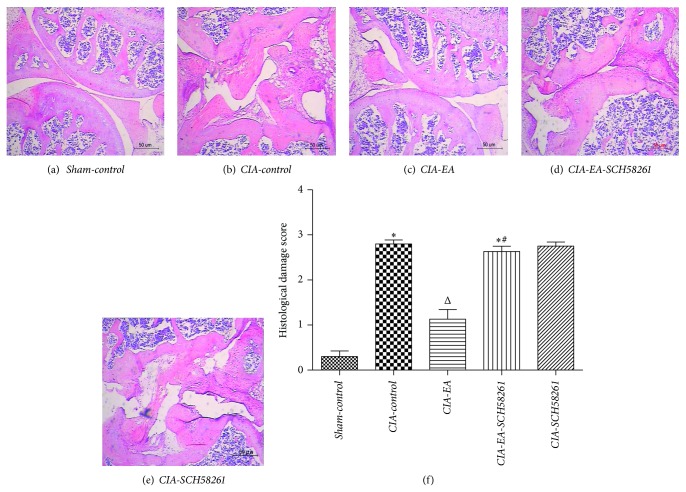
Histology of knee joints of mice with CIA. Histological evaluation of knee joints from* CIA-control* mice revealed signs of severe arthritis, with inflammatory cell infiltration, cartilage damage, and bone erosion compared to normal mice in the* Sham-control* group (a, b, and f). Histological joint alterations were significantly reduced in tissues from* CIA-EA* mice (c, f). The joint histology was not significantly different in either* CIA-EA-SCH58261* mice or* CIA-SCH58261* mice compared to* CIA-control* mice (b, d, e, and f). Values are means ± SEM of 10 animals for each group. ^*^ *P* < 0.01 versus* Sham-control*. ^Δ^ *P* < 0.01 versus* CIA-control*. ^#^ *P* < 0.01 versus* CIA-EA*. Magnification ×200.

Histological evaluation of knee joints from* CIA-control* mice revealed signs of severe arthritis, with inflammatory cell infiltration, cartilage damage, and bone erosion compared to mice in the* Sham-control* group that showed normal histology. The histological alterations apparent in joints of* CIA-control* mice were significantly reduced in* CIA-EA* mice. However, the alterations were not significantly different in either* CIA-EA-SCH58261* mice or* CIA-SCH58261* mice compared to* CIA-control* mice, and these mice also showed significantly more inflammatory cell infiltration and bone erosion than* CIA-EA* mice (Figure 4, Table 2).

### 3.4. Variation of TNF-*α* Content during Experimental Arthritis

**Figure 5:**
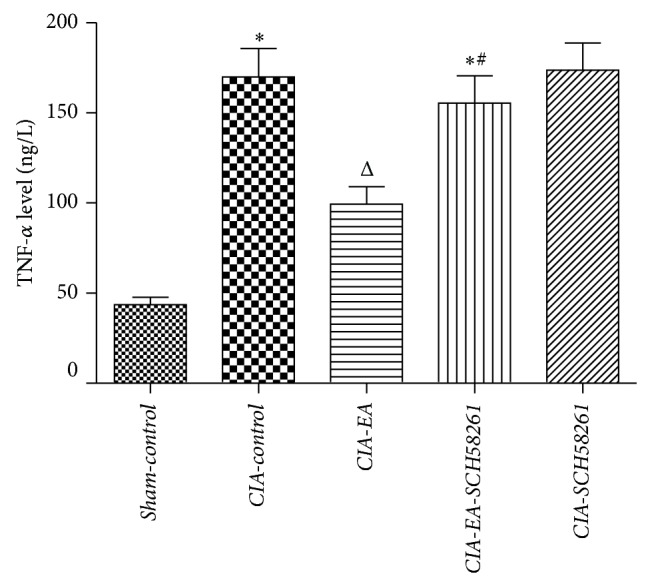
TNF-*α* plasma levels in experimental arthritis. A significant increase in plasma levels of TNF-*α* was found in* CIA-control* mice. Levels of TNF-*α* were significantly reduced in* CIA-EA* mice in comparison to* CIA-control* animals. There was no significant change in either* CIA-EA-SCH58261* mice or* CIA-SCH58261* mice in comparison to* CIA-control* animals. However, the levels of TNF-*α* in* CIA-EA-SCH58261* and* CIA-SCH58261* mice were significantly greater than in* CIA-EA* mice. Values (ng/L) are means ± SEM of 10 animals for each group. ^*^ *P* < 0.01 versus* Sham-control*. ^Δ^ *P* < 0.01 versus* CIA-control*. ^#^ *P* < 0.01 versus* CIA-EA*.

A substantial increase in production of TNF-*α* was found in CIA mice. Levels of TNF-*α* were significantly reduced in* CIA-EA* mice in comparison to* CIA-control* animals. There was no significant difference between TNF-*α* in either* CIA-EA-SCH58261* or* CIA-SCH58261* mice and* CIA-control* animals. However, the levels of TNF-*α* in both* CIA-EA-SCH58261* mice and* CIA-SCH58261* mice were significantly higher than in* CIA-EA* mice (Figure 5, Table 3).

### 3.5. Expression of A_2A_R during Experimental Arthritis

**Figure 6:**
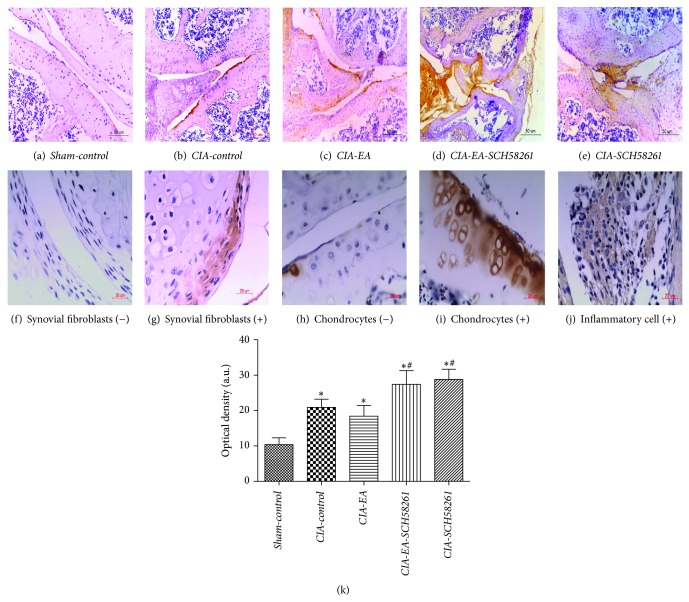
Immunohistochemical detection of A_2A_R in total ankle joints. Immunohistochemical analysis of joint sections from* CIA-control* mice revealed positive staining for A_2A_R in contrast to* Sham-control* mice (b, a), which was localized primarily in the inflammatory synovial tissue, and mainly on synovial fibroblasts (f, g), chondrocytes (h, i), and inflammatory cells (j). The staining for A_2A_R in* CIA-EA* mice was similar to that in* CIA-control* mice (b, c), while there was less inflammatory cell infiltration, synovial hyperplasia, and bone erosion (Figure 4). However, the staining for A_2A_R was significantly increased in* CIA-EA-SCH58261* mice (d) and in* CIA-SCH58261* mice (e). Values (IOD in %) are means ± SEM of 10 animals for each group. ^*^ *P* < 0.05 versus* Sham-control*. ^#^ *P* < 0.05 versus* CIA-EA* (k). Magnification ×200 (a, b, c, d, and e); ×400; (+) represented the positive staining, while (−) represented negative staining. (f, g, h, i, and j).

**Figure 7:**
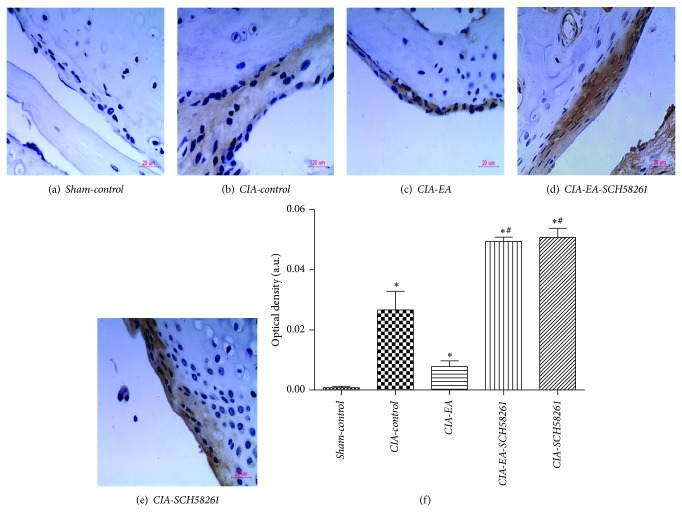
Immunohistochemical detection of A_2A_R in synovial membrane. Immunohistochemical analysis of synovial tissue from* CIA-control* mice revealed positive staining for A_2A_R in contrast to* Sham-control* mice (b, a). The staining for A_2A_R in* CIA-EA* mice was weaker than in* CIA-control* mice, but the difference was not significant (c, b). However, the staining for A_2A_R was significantly increased in* CIA-EA-SCH58261* mice (d, c) and in* CIA-SCH58261* mice compared to the* CIA-control* mice (e, b). Values (IOD in %) are means ± SEM of 10 animals for each group. ^*^ *P* < 0.05 versus* Sham-control*. ^#^ *P* < 0.05 versus* CIA-EA* (f). Magnification ×400 (a, b, c, d, and e).

In general, immunohistochemical analysis of total ankle joint sections from* CIA-control* mice revealed positive staining for A_2A_R, in contrast to* Sham-control* mice. A_2A_R was localized primarily in the inflamed synovial tissue. Firstly, A_2A_R is distributed widely on synovial fibroblasts; secondly, it is detectable in those areas infiltrated by inflammatory cells, such as macrophages, T and B cells, and neutrophils. The distribution of A_2A_R in* CIA-EA* mice was similar to* CIA-control* mice, but with less inflammatory cell infiltration, synovial hyperplasia, and cartilage destruction. However, staining for A_2A_R was significantly increased in* CIA-EA-SCH58261* mice and* CIA-SCH58261* mice compared to* CIA-control* mice and associated with more severe inflammatory cell infiltration, synovial hyperplasia, and cartilage destruction (Table 4). In addition, A_2A_R was hardly detectable on chondrocytes and only occasionally in* CIA-EA* mice (Figure 6). In synovial membrane, the overall trend of the results of the immunohistochemical analysis was similar to the results of the analysis of total joint, but the staining for A_2A_R in* CIA-EA* micewas less pronounced than in the* CIA-control* mice. However, the difference was not statistically significant (Figure 7, Table 5).

## 4. Discussion

### 4.1. Optimization of Animal Models, Acupuncture Points, and Methods

CIA is not only a widely used model of RA but is also generally used in research on anti-inflammatory and analgesic effects of acupuncture and has helped delineate the role of cellular and molecular mediators in the pathogenesis of inflammatory joint disease. There are many strains of mice or rats that can be used to induce CIA. In our study, we chose C57BL/B6 mice. 45 days after primary immunization, mice developed clinical signs of arthritis with a maximal incidence of 65% (Figure 2). Mice showed a chronic form of CIA, and this model closely resembles human RA in terms of disease course and histological findings. This is consistent with the results of Inglis et al. [18]. In addition, most transgenic and knockout strains of mice are now available on a C57BL/B6 (H-2b) background. Using the CIA model in such mice could potentially facilitate a better molecular understanding of RA. Therefore, we characterized the induction of arthritis in C57BL/B6 mice and then validated this disease model as a relevant preclinical model for RA.

EA is a modified acupuncture treatment that utilizes electrical stimulation and has been used to study the analgesic effects of EA on chronic inflammatory pain [21]. Acupoints ST36 and SP6 are frequently used in EA treatment of experimental arthritis [19, 21–23], and ST36 is the most commonly used acupoint for the purpose of immune regulation in traditional Chinese medical clinics [24]. According to traditional Chinese medical theory, the ST36 and SP6 [25] acupoints as respective representatives of the spleen and stomach meridians, are often used for disorders of the four limbs [26]. On the basis of these theories, ST36 and SP6 seemed to be the best choice for an immune-related disease with symptoms in the distal joints, such as RA.

### 4.2. Analysis of the Anti-Inflammatory Effects of EA Treatment

Several studies assessing EA treatment of RA or CIA [19, 23, 27] indicated that EA could reduce the levels of TNF-*α* in blood or synovium, inhibit the inflammatory response in joint synovial tissue, and alleviate erosion and destruction of cartilage and bone. Ouyang et al. reported [27] that TNF-*α* in blood and synovial tissue was reduced significantly after EA treatment of RA patients. Moreover, Yim et al. [19] found that EA at ST36 significantly reduced interleukin-6 (IL-6), TNF-*α*, interferon-*γ*, anti-collagen II antibody, and IgG and IgM levels in serum of CIA mice and prevented knee joint destruction.

In good agreement with these studies, we observed lower TNF-*α* levels, which correlated with reduced paw swelling, lower clinical scores, less histological damage, and radiographic bone erosion in* CIA-EA* mice than in* CIA-control* mice. Significantly, there was no significant change of the above-mentioned indices in* CIA-SCH58261* mice compared to* CIA-control* mice. This suggests that the A_2A_R antagonist SCH58261 itself does not affect the severity of CIA. In contrast, EA treatment resulted in a statistically significant decrease in TNF-*α* levels, paw swelling, clinical scores, and the histological/radiographic severity of the disease, compared to* CIA-control* mice. This strongly suggests that EA treatment using ST36 and SP6 exerted an anti-inflammatory effect in CIA.

### 4.3. Mechanism of A_2A_R in EA Treatment on CIA

Varani et al. showed [28] that A_2A_R was upregulated in lymphocytes from early RA patients and A_2A_R activation inhibited the NF-kB pathway and decreased the expression of inflammatory cytokines such as TNF-*α*, IL-1*β*, and IL-6. A_2A_R and A3R density inversely correlated with DAS28 and DAS, indicating an association between receptor expression and severity of joint inflammation in RA. These phenomena could be reversed by A_2A_R antagonists. In addition, de Mendonça et al. found that, in lipopolysaccharide-activated monocytes, inhibition of A_2A_R activation resulted in increased TNF-*α* secretion, while the activation of A_2A_R resulted in decreased TNF-*α* secretion. These studies also demonstrated that A_2A_R is the main adenosine receptor affecting the secretion of TNF-*α* by monocytes [29]. Moreover, Mediero et al. found that A_2A_R agonists inhibited differentiation and function of osteoclasts and reduced IL-1*β* and TNF-*α* secretion and that this inhibition could be reversed by A_2A_R antagonists [30]. Recently, Andreas et al. [31] defined some key regulatory molecules of cartilage destruction in RA; among them, A_2A_R is an important immunomodulator of inflammation.

In the current study, we found that A_2A_R was localized primarily in the inflamed synovial membrane of CIA mice on RASF and infiltrating inflammatory cells, but it was hardly detectable on chondrocytes, only occasionally in* CIA-EA* mice. A_2A_R expression in the total ankle joint in* CIA-control* mice was upregulated, while it showed a tendency of reduced expression after EA treatment. Detailed analysis of the A_2A_R immunoreactivity in synovial membranes indicated that the A_2A_R expression pattern in RASF and infiltrating inflammatory cells was consistent with that of the whole joint.

Administration of the A_2A_R antagonist SCH58261 resulted in increased expression of A_2A_R that was independent of EA treatment. Simultaneously, the anti-inflammatory effect of EA was completely abolished by administration of the A_2A_R antagonist, suggesting that A_2A_R signaling is involved in and essential for the anti-inflammatory effect of EA.

The observed pattern of A_2A_R expression is consistent with a negative feedback mechanism of A_2A_R signaling on A_2A_R expression. A_2A_R signaling induced by EA resulted in A_2A_R downregulation, while suppression of A_2A_R signaling by the antagonist SCH58261 resulted in strong upregulation of A_2A_R expression in the presence of local inflammation. However, this upregulation of A_2A_R expression in the absence of A_2A_R signaling failed to mediate the anti-inflammatory effects of EA.

This suggests that EA exerts its anti-inflammatory effect though local elevation of adenosine and subsequent signaling through A_2A_R. Local elevation of adenosine levels at acupuncture sites has been described [9]. The mechanism of local adenosine increase at the acupuncture site still needs to be elucidated by future research.

## 5. Conclusions and Outlook

Our experimental data suggest that EA treatment exerts an anti-inflammatory effect and effectively protects against CIA-induced joint damage, but A_2A_R antagonists reversed this effect. Thus, the A_2A_R may mediate the anti-inflammatory and tissue-protective effects of EA. Additional studies, including the use of A_2A_R gene knockout animal models, will be needed to conclusively elucidate the mechanism of action of EA in order to justify its use in the treatment of human RA.

## Figures and Tables

**Table 1:** Radiographic scores of mice in the different treatment groups.

Variable	*n*	Radiographic score
*Sham-control *	10	0.20 ± 0.26
*CIA-control *	10	2.50 ± 0.41^*^
*CIA-EA *	10	1.05 ± 0.50^△^
*CIA-EA-SCH58261 *	10	2.35 ± 0.47^∗#^
*CIA-SCH58261 *	10	2.50 ± 0.50

^*^ *P* < 0.01 versus *Sham-control*. ^△^ *P* < 0.01 versus *CIA-control*. ^#^ *P* < 0.01 versus *CIA-EA*.

**Table 2:** Histological damage scores of mice in the different treatment groups.

Variable	*n*	Histological damage score
*Sham-control *	10	0.30 ± 0.40
*CIA-control *	10	2.80 ± 0.28^*^
*CIA-EA *	10	1.13 ± 0.67^△^
*CIA-EA-SCH58261 *	10	2.63 ± 0.37^∗#^
*CIA-SCH58261 *	10	2.75 ± 0.29

^*^ *P* < 0.01 versus *Sham-control*. ^△^ *P* < 0.01 versus *CIA-control*. ^#^ *P* < 0.01 versus *CIA-EA*.

**Table 3:** TNF-*α* plasma levels in mice in the different treatment groups (ng/L).

Variable	*n*	TNF-*α*
*Sham-control *	10	43.60 ± 12.99
*CIA-control *	10	169.98 ± 49.84^*^
*CIA-EA *	10	99.47 ± 30.44^△^
*CIA-EA-SCH58261 *	10	155.29 ± 48.28^∗#^
*CIA-SCH58261 *	10	173.76 ± 47.57

^*^ *P* < 0.01 versus *Sham-control*. ^△^ *P* < 0.01 versus *CIA-control*. ^#^ *P* < 0.01 versus *CIA-EA*.

**Table 4:** A_2A_R expression (integrated optical density) in ankle joints of mice in the different treatment groups (IOD in %).

Variable	*n*	Optical density
*Sham-control *	10	10.39 ± 6.06
*CIA-control *	10	20.90 ± 7.03^*^
*CIA-EA *	10	18.41 ± 9.53^*^
*CIA-EA-SCH58261 *	10	27.42 ± 12.81^∗#^
*CIA-SCH58261 *	10	28.81 ± 9.05^∗#^

^*^ *P* < 0.05 versus *Sham-control*. ^#^ *P* < 0.05 versus *CIA-EA*.

**Table 5:** A_2A_R expression (integrated optical density) in synovial membranes of mice in the different treatment groups (IOD in %).

Variable	*n*	Optical density
*Sham-control *	10	0.0008 ± 0.00143
*CIA-control *	10	0.0266 ± 0.01948^*^
*CIA-EA *	10	0.0078 ± 0.00604^*^
*CIA-EA-SCH58261 *	10	0.0493 ± 0.00482^∗#^
*CIA-SCH58261 *	10	0.0507 ± 0.00965^∗#^

^*^ *P* < 0.05 versus *Sham-control*. ^#^ *P* < 0.05 versus *CIA-EA*.
